# Monomyeloid precursors in drug-induced hypersensitivity syndrome

**DOI:** 10.1186/2045-7022-4-S3-P146

**Published:** 2014-07-18

**Authors:** Hideo Hashizume

**Affiliations:** 1 Department of Dermatology, Shimada Municipal Hospital, Japan

## 

Drug-induced hypersensitivity syndrome (DIHS), also known as drug reactions with eosinophila and systemic symptoms, has unique clinical features including fever, generalized rash, lymphadenopathy and extracutaneous organ involvements. During the disease course, human herpesvirus (HHV)-6 reactivation is frequently (60%<) observed in association with unfavorable outcomes. We recently noticed a transient increase of circulating monocyte-sized cells that showed higher side scatter counts in flowcytometer analysis before HHV-6 reactivations. These cells were CD14lowCD16+, like a minor (<10%) monocyte subset that differentiates into 'resident' dendritic cells/macrophages, but were CD4- CD11a- CD11b+ CD13int CD34- CD41ab- CD66b- CD117- CD163- and contained rich organelles histologically, suggesting mono-myeloid precursors, not bona fide monocytes. We also found HHV-6 genome and antigens in them. Since mono-myeloid cells in the bone marrow are reportedly reservoirs of HHV-6, the mono-myeloid precursors that emerged transiently in circulation in DIHS patients might emigrate from the bone marrow, and are suggested to play a special role of the virus reactivation. Our observations will provide a scenario that circulating mono-myeloid precursors bridge between drug allergy and viral infection, which remains poorly understood, in DIHS.

